# Comparison of fracture resistance of endodontically treated teeth using different coronal restorative materials: An *in vitro* study

**DOI:** 10.4103/0972-0707.58338

**Published:** 2009

**Authors:** Prashant Monga, Vivek Sharma, Sukesh Kumar

**Affiliations:** Departments of Conservative Dentistry and Endodontics, D.J. College of Dental Sciences and Research, Modinagar, U.P, India

**Keywords:** Bonded restorations, fracture resistance, root-filled teeth

## Abstract

**Aim/Objective:**

To evaluate the *in vitro* effect of bonded restorations on the fracture resistance of root canal-treated teeth.

**Materials and Methods:**

One hundred twenty extracted, maxillary, permanent premolars were collected. After preparing the access cavity, the teeth were biomechanically prepared and obturated. Samples were divided into six groups based on the type of restorative material used to restore them. Teeth were embedded in acrylic resin and their fracture strength was measured using a Universal Testing Machine. Data were evaluated statistically using one-way ANOVA-F and unpaired t-test.

**Results:**

Teeth restored with bonded amalgam and composite resin showed higher fracture resistance than those restored with conventional amalgam. Fracture strengths of bonded restorations and intact teeth were not statistically different. The results suggested that the group restored with conventional amalgam had the lowest fracture resistance. No statistically significant differences were found between the bonded amalgam and composite resin groups.

**Conclusion:**

Conventional amalgam core showed the least fracture resistance whereas; composite resin and bonded amalgam core showed fracture resistance was similar to that of natural tooth.

## INTRODUCTION

Restoration of root canal-treated teeth with a permanent, definitive, postendodontic restoration is a final step for successful root canal treatment as these teeth are considered more susceptible to fracture. The reason most often cited for this finding has been the dehydration and loss of dentin after the endodontic procedures and the removal of important anatomic structures such as cusps, ridges, and the arched roof of the pulp chamber, all of which provide much of the necessary support for the natural tooth.[1]

Therefore, intracoronal strengthening of teeth is important to protect them against fracture, particularly in posterior teeth where stresses generated by occlusal forces can lead to fracture of unprotected cusps. Restoration of root canal-treated teeth is an important step that complements a technically sound endodontic treatment.[2] Thus, root canal treatment should not be considered complete until a coronal restoration has been placed. An optimal final restoration for endodontically treated teeth maintains aesthetics, function, preserves the remaining tooth structure, and prevents microleakage.[3]

Studies suggest that complex amalgam restorations, complete cast coverage, cast restorations, and composite materials can all be used as postendodontic restorations. Although dental amalgam has favorable mechanical properties, it lacks adhesion to the tooth structure. This diminishes the fracture resistance of the remaining tooth structure due to microcrack propagation under fatigue loading.[4] Cast restorations and complete cast coverage procedures involve multiple visits and increased cost, which can lead to increased chances of discontinuation of the treatment.[5,6]

With the recent advancements in adhesive technology and stronger adhesive materials, it is now possible to create conservative, highly aesthetic restorations[7] that are bonded directly to the tooth structure and strengthen it.[8]

Introduction of new bonding agents has also led to the possibility of restoring root-filled teeth with a bonded restoration instead of a crown or onlay restoration.[2] The ability to predictably restore a root-filled tooth to its original strength and fracture resistance without the placement of a full coverage restoration could provide potential prosthodontic and economic benefits to patients.

The aim of the study was to evaluate the *in vitro* effect of bonded restorations on the fracture resistance of root canal-treated teeth.

## MATERIALS AND METHODS

One hundred twenty freshly extracted, intact, noncarious, human, maxillary, premolar teeth with similar anatomic characteristics were selected. All soft tissue and debris on the teeth were removed using an ultrasonic scaler and the teeth were stored in saline at room temperature. To minimize the influence of size and shape variations on the results, the teeth were classified according to their mesiodistal and buccolingual dimensions. The teeth were randomly divided into six experimental groups of 20 teeth each and subjected to the following procedures:

Group 1 – Unaltered teeth (control)

Group 2 – Standard endodontic access cavities were prepared with No. 245 straight burs

Group 3 – MOD (mesial-occlusal-distal) cavities were prepared with airotor no. 245 straight burs so that the buccolingual width of the occlusal isthmus was one third the width of the intercuspal distance, and the buccolingual width of the approximal preparation was one third of the buccolingual width of the crown. The approximal boxes were prepared straight (nonundercut) and limited to 2 mm coronally in the depth from the cemento-enamel junction.

An endodontic access cavity was then prepared; the root canals instrumented to a size 40 file (Mani, Inc, Tochigi, Japan) and filled with gutta-percha (SPI Dental Mfg. Inc., Inchon, Korea) and AH 26 root canal sealer (Dentsply DeTrey, Konstanz, Switzerland) using a lateral condensation technique.

Group 4 – The teeth were prepared and the root canals were filled as in group 3. Cavities were restored conventionally with high-copper amalgam [Dispersalloy, Dentsply] [Table 1] according to the manufacturer's instructions.

**Table 1:** Restorative materials used

Restorative material	Composition	Manufacturer
Dispersalloy	High-copper amalgam	Dentsply Int. Ltd.Maillefer, USA
Adper scotchbond Multi-Purpose plus Adhesive system	Activator which is an ethanol-based solution of a sulfinic acid salt and a photoinitiator component Primer is an aqueous solution of HEMA and a polyalkenoic acid copolymer Adhesive is a BIS-GMA and HEMA resin combined with a novel initiation system Catalyst is comprised of the same BIS-GMA and HEMA resin system but incorporates the peroxide component of a self-cure resin system	3M ESPE, ST Paul, USA
Filtec P60	Posterior composite	3M ESPE, STPaul, USA

Group 5 – The teeth were prepared and the root canals were filled as in group 3. Prior to the restoration with amalgam, the Adper Scotchbond Multi-Purpose plus Adhesive system (3M ESPE) was applied according to the manufacturer's instructions. Etchant (37% Phosphoric acid) was applied to the enamel and dentine for 15 seconds. The cavity was rinsed and excess water removed with a gentle, five-second air blast. One drop each of activator (bottle 1.5) and primer (bottle 2) were mixed and applied to the etched enamel and dentine for 15 seconds; the preparations were dried gently for five seconds. One drop each of adhesive (bottle 3) and catalyst (bottle 3.5) were then mixed and applied to the primed enamel and dentine. The amalgam was mixed and placed before the bonding material had set; the restorations were then polished.

Group 6 – The teeth were prepared and the root canals were filled as in group 3. Prior to the restoration with composite resin, the Adper Scotchbond Multi-Purpose plus Adhesive system (3M ESPE) was applied according to the manufacturer's instructions. Both enamel and dentine were etched with 37% phosphoric acid for 15 seconds. The surface was rinsed with water and the excess water was removed with an air syringe. Scotchbond multipurpose primer (bottle 2) was applied to the enamel and dentine and was dried gently for five seconds. Adper Scotchbond Multi-Purpose plus Adhesive was then applied to the enamel and dentine and light-cured for ten seconds. The composite resin (Filtec P60, 3M ESPE) was placed in the cavities in increments of 2 mm thickness, and each increment was light-cured for 20 seconds. After the removal of the matrix band, the restorations were contoured and polished.

Teeth were stored in 100% humidity at 37° C for seven days. Cylindrical moulds (2.5 cm × 2.5 cm) were made using stainless steel pipes. Self-cure acrylic resin [Ashwin Pvt. Ltd. India] was used to fill the mould and the teeth mounted to 1 mm level apical to the cemento-enamel junction.

Fracture strength testing was done using a Universal Testing machine [Llyold, UK]. Prepared specimens were then mounted on a holder slot which was fixed to the lower arm of the universal testing machine. A metal indenter of 6 mm diameter was fixed to the upper arm of the universal testing machine which was set to deliver an increasing load until fracture occurred.

The crosshead speed was 1.0 mm per minute, and the load was applied to the occlusal inclines of the buccal and lingual cusps vertically down the long axis of the tooth. The force required to fracture each tooth was recorded in Newtons. Statistical analysis was performed using one way ANOVA-F test and unpaired *t*-test to determine the significance of differences between different groups.

## RESULTS

The mean forces at fracture, the minimal and maximal values and the SD for each group are presented in Table 2. The mean forces at fracture were: Group 1 (1193.75 N), group 5 (968.00 N), group 6 (867.38 N) followed by group 2 (683.10 N), group 4, (501.10 N) and group 3 (248.50 N), respectively. The overall significant difference between the groups was found at the 0.01 level (*P* < 0.01). According to the unpaired *t*-test results, significant differences were found between the teeth restored with conventional amalgam or bonded amalgam (groups 4 and 5, *P* < 0.01) and those restored with conventional amalgam and composite resin (groups 4 and 6, *P* < 0.01). There were no significant differences between unaltered teeth and bonded amalgam groups (groups 1 and 5) or between groups 2 and 4. In addition, no statistically significant difference was found between the bonded amalgam and composite resin groups (groups 5 and 6). The mean force at fracture in group 3 (MOD plus access cavity) was significantly lower than in the other groups (*P* < 0.01). Groups with significant difference are shown with different superscripts in Table 2.

**Table 2:** Forces at fracture points in root canal-treated teeth restored with different materials

Groups	n	Mean ± SD	Minimum	Maximum
1^a^	20	1193.75 ± 365.72	441.00	1860.00
2^b^	20	683.10 ± 221.34	424.00	1085.00
3^d^	20	248.50 ± 110.75	110.00	640.00
4^b^	20	501.10 ± 203.56	258.00	975.00
5^a,c^	20	968.00 ± 186.41	534.40	1475.20
6^c^	20	867.38 ± 218.90	388.00	1260.00

Mean, minimal, and maximal forces at fracture values (Newtons) and SDs. Groups with no statistical difference are shown with the same superscripts

## DISCUSSION

Tooth restoration is the final step in root canal treatment.[5] Numerous studies have been conducted to determine the ideal method to restore endodontically treated teeth as these teeth have decreased fracture resistance due to the loss of tooth structure during endodontic access and cavity preparation procedures. Cusp separation rarely occurs in noncarious, intact teeth because of the presence of the pulp chamber's roof and marginal ridges, which can be considered to be tooth-reinforcing structures. The presence of palatal and buccal cusps with intact mesial and distal marginal ridges forms a continuous circle of tooth structure which reinforces and maintains the integrity of the tooth.[8]

It has been shown that the weakening of teeth due to restorative and endodontic procedures increases with the reduction of tooth structure.[4,2] Endodontic procedures reduce the relative rigidity of the tooth by 5%, which is contributed entirely by access opening. In contrast, loss of the marginal ridge has resulted in a 46% loss in tooth rigidity whereas an MOD preparation has resulted in a loss of 63% relative cuspal rigidity.[9] Other authors reported that the mean fracture strength for unrestored teeth with MOD preparation was 50% less than that of unaltered premolar teeth.[5] It was observed that cavity preparations made with occlusal opening and marginal ridge removal resulted in elevated strain values, supporting the premise that teeth are weakened by the removal of tooth structure.[8] This highlights the importance of prevention and early diagnosis of carious lesions before they involve the marginal ridge.

Traditionally, root canal-treated teeth have been restored with cast restorations and full/partial coverage crowns which include cusp coverage to improve the fracture resistance.[9] To further increase the fracture resistance, several attempts have been made to restore endodontically treated teeth with different post systems to increase the fracture resistance of the root structure. However, some studies have proved that these posts decrease the fracture resistance instead of increasing it. Endodontic posts do not reinforce the crown as enlargement of the root canal space after completion of root canal treatment can weaken the tooth structure. Another method that has been used is cusp reinforcement with the use of pins. Although restored teeth can be as strong as intact teeth, these pins create stress and suffer corrosion in the dental tissue.[10]

Numerous materials have been used as substitutes for dental tissues. Amalgam, for instance, is the most common material used for more than 100 years in posterior restorations. Although amalgam has high compressive strength, it does not adhere to the dental structure. Cuspal fractures in amalgam restoration result from the fatigue caused by crack diffusions subjected to repeated loading. Also, the presence of mercury and the types of interactions among its metal components make this material exhibit higher deformation levels when submitted to occlusal load application.[8]

In the recent past, the introduction of new bonding agents with bond strength between 20 and 25 Mpa (and sometimes even more) has led to the suggestion that endodontically treated teeth may be restored with a bonded restoration instead of a crown or onlay preparation. These bonding agents have the advantages of providing greater strength and fracture resistance without the placement of full-coverage restoration and also limiting coronal microleakage which may emerge as an important factor in the selection of bonding agents for the coronal buildup of endodontically treated teeth.[11] These qualities provide potential prosthodontic and economic benefits to patients.[10] For these reasons, the use of adhesive materials has been considered useful for tooth reinforcement. These observations have been confirmed by other studies also.[12]

Teeth restored with amalgam bonded to etched enamel have a significantly greater fracture resistance than those restored with conventional amalgam due to its adhesion to the tooth structure. Adhesion occurs between the amalgam and the tooth structure through the use of the bonding agent between them. The adhesive resin develops mainly micromechanical retention with the tooth structure. It may also develop some chemical bonds via phosphate esters that interact with calcium ions in the tooth. When the amalgam is packed into the cavity over the wet resin, a mechanical interlocking of the resin and the amalgam occurs. This interlocking is probably a much more significant factor in the retention of the amalgam than are the chemical bonds that occur between the resin and the components of the amalgam.[13]

In addition to aesthetics, modern composite materials have got high compressive strength for posterior restorations. It has been suggested that the use of resin composite in restorations reinforces dental stiffness as the adhesive nature of the composite binds the cusps and decreases their flexion. Flexion is considered to be the main cause of fracture in conventional, nonbonded amalgam restorations. Due to its low elastic modulus, composite resin can transmit the energy produced by the compressive forces to the adjacent dental structure, thus reinforcing the weakened tooth structure. Although, the tooth restoration interface suffers elastic stresses generated by the contraction of the material during polymerization, these stresses can be dissipated by cuspal movement.[12]

In our study, conventional amalgam, bonded amalgam, and composite resin were used to restore endodontically treated maxillary premolars. Maxillary premolars were used because studies have shown that these teeth are more prone to fracture. Reasons cited for this greater susceptibility to fracture are: i) the anatomical shape of maxillary premolars that creates a tendency for the separation of their cusps during mastication. Clinically restored maxillary premolars may undergo palatal and buccal strain as a result of occlusal load application, which may be associated with high levels of stress concentration inside the tooth restoration complex. The cusp inclines of these teeth are much greater than in maxillary molars and can result in a different pattern of fracture resistance. It has also been reported that the incidence of fracture is greater in maxillary premolars than in mandibular premolars. The lowest 20 years' survival rate was found in maxillary premolars.[13] ii) Other authors have noted a difficulty in obtaining uniform fracture strengths for human teeth due to natural variations in tooth morphology.[14,15] Maxillary premolars were selected as it is known that they show the least variations. iii) Direct composite restoration of premolars can be considered to be more predictable than that of molars due to the small amount of composite needed for restoration which, in turn, leads to lower polymerization contraction stress. iv) Also, the interproximal margins of premolars are more accessible for inspection and finishing procedures.[16]

Mesio-occlusal distal (MOD) cavities were prepared in our study to simulate this preparation that is often found clinically and has been extensively reproduced in other clinical studies. The general effect of MOD cavity preparations is the creation of long cusps; thus, there is the need for a restorative material that not only replaces the lost tooth structure, but also increases the fracture resistance of the residual tooth and promotes effective marginal sealing.

Several studies have shown that applying the force to the long axis of the, tooth transmits the force uniformly.[17–20] In our study, force was also applied vertically at a constant speed using a universal testing machine. When the cylindrical-shaped tool makes contact with the tooth, it acts as a wedge between the buccal and lingual cusps in unrestored teeth and decreases the mean fracture resistance values, while promoting more catastrophic types of fracture. Our research evaluated the capacity shown by the material to support vertical tension, vital in areas of high masticatory effort.

In our study, the highest mean fracture value was found in intact teeth (Group 1) because there was no loss of tooth structure.

The difference between groups 5 and 1, *i.e*., the bonded amalgam and intact teeth groups was not statistically significant. These findings may be related to the reinforcement of tooth structure due to adhesion of the amalgam, thus increasing the fracture resistance. These findings are in accordance with other studies that found that bonding amalgam to the tooth structure not only increased fracture resistance of teeth, but could also restore the strength and rigidity lost by cavity preparation.[21,22] It was also found that teeth restored with bonded amalgam were significantly more resistant to fracture than those restored with conventional amalgam.[19,23,24]

The fracture resistance of group 6 (restored with composite resin) was not significantly different from group 5 (restored with bonded amalgam). These findings can again be related to the adhesive nature of composite resin to the tooth structure and the increased strength of newer posterior composites available in the market. These findings are in accordance with similar studies conducted in the recent past that found that endodontically treated teeth restored with composite resin had higher fracture resistance than those restored with nonadhesive restorations.[25,26] When the clinical success rates of endodontically treated premolars restored with direct composite restorations were evaluated after three years of service, they were found to be equivalent to a similar treatment of full coverage with metal ceramic crowns.[27] Thus, composite resin plays an important role in recovering tooth strength.[8]

Group 4 (restored with conventional amalgam) exhibited significantly lower fracture resistance than groups 5 and 6 (*i.e*., bonded amalgam group and composite resin group respectively) probably due to the fact that conventional amalgam did not adhere to the tooth structure. These findings are in accordance with similar studies that compared the stiffness of endodontically treated premolars, and found that teeth restored with amalgam were the weakest whereas those restored with composite restoration were as strong as unaltered teeth. The results of our study suggest that adhesion plays an important role in increasing the fracture resistance of endodontically treated teeth.[24,28]

We suggest that bonded composite resin restoration can be considered as the first choice for aesthetic reasons. However, if for some reason, the clinician chooses amalgam, bonding amalgam to tooth structure could be expected to produce a higher fracture resistance compared with a conventional amalgam restoration.

We are in the process of carrying out additional clinical studies to determine the long-term, *in vivo* prognosis of extensive bonded restorations in endodontically treated teeth. More studies involving the use of adhesive restorative materials in restoring root-filled teeth are required.

## CONCLUSIONS

1. The teeth restored with conventional amalgam were significantly weaker than the teeth restored with bonded amalgam and composite resin (*P* < 0.01).
2. No statistically significant differences were found between the bonded amalgam and composite resin groups or between the bonded amalgam group and sound teeth.
